# Should newborn genetic testing for autism be introduced?

**DOI:** 10.1136/jme-2024-110166

**Published:** 2024-12-03

**Authors:** Ramkumar Aishworiya, Hui-Lin Chin, Julian Savulescu

**Affiliations:** 1 Division of Developmental and Behavioural Paediatrics, Department of Paediatrics, Khoo Teck Puat – National University Children’s Medical Institute, National University Hospital, Singapore; 2 Department of Paediatrics, Yong Loo Lin School of Medicine, National University of Singapore, Singapore; 3 Division of Genetics and Metabolism, Department of Paediatrics, Khoo Teck Puat – National University Children’s Medical Institute, National University Hospital, Singapore; 4 Centre for Biomedical Ethics, Yong Loo Lin School of Medicine, National University of Singapore, Singapore; 5 Oxford Uehiro Centre for Practical Ethics, Faculty of Philosophy, University of Oxford, Oxford, UK

**Keywords:** Ethics, Infant- Newborn, Genetic Carrier Screening

## Abstract

This manuscript provides a review of the potential role of newborn genetic testing for autism, and whether the state has an inherent responsibility to facilitate and subsidise this. This is situated within the broader construct of benefits and limitations of genetic testing currently. Potential benefits of such presymptomatic genetic testing include facilitating earlier diagnosis and access to appropriate intervention which can improve the treatment outcome for the child and indirectly benefit caregivers and society by reducing the care needs of the child and adult in future. However, there are several limitations to newborn genetic testing including the variable penetrance of ‘autism-risk’ genes, marked phenotypic heterogeneity of autism, real-world limitations in access to treatment, potential psychological harm to caregivers and financial considerations. We hence argue for facilitation of diagnostic genetic testing instead, especially for parents who seek to have greater understanding of recurrence likelihoods, related to reproductive decision-making. Facilitation of such testing can be in the form of both financial subsidies and infrastructural elements including availability of testing facilities and trained healthcare personnel for individualised pregenetic and postgenetic test counselling.

## Case

 J and P are a young married couple and parents of B, their toddler child. B has age-appropriate motor skills but is non-verbal and socially withdrawn with global developmental delay. B has just been diagnosed to be on the autism spectrum by his developmental paediatrician. J and P are asked to decide on whether they would like B to undergo whole exome sequencing in order to try to ascertain a potential genetic cause for his autism. The test will cost approximately US$3500 which is a significant financial burden for the couple. However, they are keen to know if there is a specific genetic mutation in B, especially because they are contemplating having another child in the near future and worry about whether he/she will be on the autism spectrum as well. Eventually, they decline the test, due to financial difficulties and the need to provide for behavioural therapy for B. The couple remain extremely concerned about the chance of their next child being on the autism spectrum.

Should the healthcare system subsidise the cost of genetic testing for children on the autism spectrum? Further, should genetic testing for autism be included as part of newborn genetic screening, and if so, what role does the healthcare system have towards this?

## Introduction

The current century of medicine has seen autism receive the limelight as the leading neurodevelopmental disorder of childhood. Autism is characterised by difficulties with social communication occurring together with behavioural impairments such as rigidity, repetitive movements and sensory preferences.^1^ This condition is becoming increasingly prevalent, with global median prevalence of 1 in 100 children and estimates from some countries such as the US citing rates as high as 1 in 36 children.^2 3^ One of the hallmarks of the condition has been the broad and varying degree of impairments among afflicted individuals. While there is growing recognition of those with minimal support needs, who are cognitively able and functioning independently within society, there continues to be a significant proportion of individuals with what has now been termed ‘profound autism’—these children and adults have significantly impaired communication skills, often being minimally verbal and almost always having co-occurring intellectual disability.^4^

Much of research in the scientific field of autism has focused on the medical aspects, including understanding its biological basis and subtypes and advancing treatments for children on the autism spectrum, encompassing both behavioural interventions and medications. An important aspect of this pursuit has been the field of autism genetics. We now know that the aetiology of autism is likely a complex interplay of an underlying genetic susceptibility with epigenetic and environmental factors.^5 6^ Twin and sibling studies have shown that the chance of having autism is 10%–20% in someone with a sibling on the autism spectrum and this increases to almost 90% in monozygotic twins and around 60% in dizygotic twins.^7^ Several large-scale research studies over the last 20 years have identified several ‘autism-risk genes’ which have been associated with a higher likelihood of autism.^8 9^ It is likely that the presence of such genes leads to an increased genetic predisposition to autism either by themselves or in conjunction with as yet undefined environmental factors, which interact to eventually lead to the autism phenotype. Such genetic variants may arise de novo in the child, be inherited from carrier parents for X-linked or autosomal recessive disorders or be inherited from minimally/apparently unaffected parents for autosomal dominant conditions. Although many of these autism-risk genes are associated with a high chance of having the autism phenotype, collectively these account for only a relatively small proportion of cases of autism. Likewise, the presence of many of these genetic variations does not always guarantee the presence of autism.^10^

Given this growing scientific knowledge, genetic testing with the aim of identifying a specific genetic cause has become a standard part of clinical care for children following an autism diagnosis in some countries.^11^ Chromosomal microarray was previously considered the standard initial genetic test with a diagnostic yield of approximately 12.2%.^12^ To date, there are at least 1586 genes annotated to associate with autism,^13^ thus the significant genetic heterogeneity for this necessitates broad approaches to genetic testing. Exome sequencing has been shown to have a diagnostic yield of up to 36%,^14^ and genome sequencing up to 68%,^15^ and thus has replaced the microarray as the recommended first-tier test for children with neurodevelopmental disorders.^10 14^ Panel analysis comprising a selection of autism-associated genes is also available, though the yield of these are variable and depend on the coverage of genes included in the analysis.

Newborn screening refers to a set of tests performed on the newborn to evaluate the newborn for diseases. These typically include biochemical tests, for example, measurement of thyroid stimulating hormone for congenital hypothyroidism, measurement of G6PD activity for glucose-6-phosphate dehydrogenase deficiency, tandem mass spectrometry for biochemical parameters suggestive of inborn errors of metabolism like phenylketonuria, or physical tests, for example, hearing screen for congenital hearing loss. Diseases included in newborn screening vary amongst healthcare systems depending on disease prevalence, funding availability and systemic priorities. Selection of disorders suitable for newborn screening is usually guided by the 1968 Wilson and Jungner criteria.^16 17^ Newborn genetic screening using genetic tests like gene panel analysis, exome sequencing or genome sequencing is increasingly being considered and piloted in healthy newborns for early-onset genetic disorders to facilitate prevention and early treatment.^18^,^21^ Genes included in prior newborn genetic screening studies have been heterogeneous, including inborn errors of metabolism, neurological disorders like epilepsy and genes for other genetic disorders involving different body systems. None have focused on inclusion of autism. There is a need for overall consensus on genes and conditions included, a need to evaluate economic, ethical and psychosocial impacts, and to build infrastructure and develop management guidelines for presymptomatic care of included conditions for broader implementation.^22^

**Figure 1:**
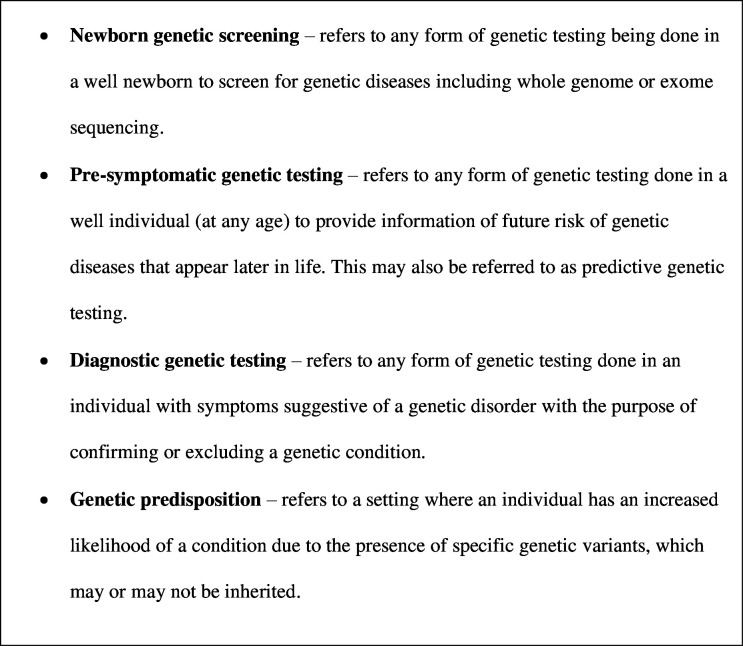
Definitions of specific terms used.

Given such advances, is there a role for including analysis for autism in newborn genetic screening? And if so, how much of a role does the state have to facilitate and/or subsidise such screening? Can this help with early identification of children who need autism-specific intervention? In this paper, we examine the arguments for and against the inclusion of autism-specific genetic testing for all newborns, within a healthcare system that subsidises such testing to some extent and compare diagnostic versus presymptomatic testing. Figure 1 provides definitions of the specific terms used in this manuscript.

### Potential benefits of newborn genetic screening for autism

The main argument for newborn genetic screening for autism would be the early identification of a child on the autism spectrum. This is in keeping with beneficence to the child and centres around the premise that current medical diagnostic tools in autism are designed to determine diagnosis only in toddlerhood, from around 18 months of age and at best after 12 months of age. This often leads to treatment initiation after diagnosis, typically between 2 and 3 years of age. In many communities, average age of diagnosis is in fact much later, occurring between 3 and 4 years leading to treatment initiation at an even older age.^23^ However, current behavioural interventions for autism are more successful when they are initiated at a younger age, in keeping with the concepts of neuroplasticity.^24 25^ Thus, one can argue that identifying children at higher likelihood of autism at a younger age—for example, soon after birth—enables for presymptomatic intervention for autism, which has the potential to improve outcomes in these individuals. Presymptomatic intervention of infants at higher likelihood from an early age has been shown to reduce the odds of attaining an autism diagnosis at 3 years in a previous randomised controlled trial (6.7% in intervention group vs 20.5% in control group, OR 0.18 for intervention).^26^ Thus, there is significant interest to move towards even earlier diagnosis and intervention.^27 28^

Another potential benefit is that of beneficence to parents and society—whereby, presymptomatic intervention can result in improved development, and hence the responsibilities of care for parents/caregivers can be reduced.^29^ Parents of children on the autism spectrum are known to have high levels of stress and anxiety.^30 31^ The child’s degree of difficulties resulting from autism and its related behaviours have been known to be factors associated with this stress.^32^ Thus, improved child outcomes can translate to lesser care responsibilities for caregivers. In turn, at a societal level, this may lead to adults on the autism spectrum who are better able to independently function within society and contribute to it better.

### Potential harms of routine newborn genetic screening

There are several problems with the basic premise that newborn genetic screening can lead to earlier identification and treatment for children on the autism spectrum. First, genes associated with autism can have variable expression and penetrance, such that the presence of a genetic variant does not necessarily guarantee the future development of autism.^33^ Whether autism will be present, what degree of impairments it will entail and from which age point would the child benefit from early intervention are all important but unanswered questions. Some genes such as *MBD5*, *BCKDK*, *FOXG1*, *MECP2*, *TBC1D20* have full penetrance and autism expression.^34^ It may be reasonable to include testing for established genes with full penetrance and expression of autism in newborn genetic screening. Others may have highly variable phenotypes that include autism, variable intellectual disability and epilepsy risk. The recurrent 22q11.2 duplication is an example of a genetic finding with highly variable risk for autism and other comorbidities. Autism also has a large phenotypic variance, such that genetically predisposed individuals may be on the spectrum but have low support needs.^35^ Such an individual may not necessarily need behavioural intervention in childhood and could only possibly have evolving needs for intervention and support in later years. This complicates newborn genetic screening in terms of what findings to report and how to execute post-test genetic counselling to help the family make sense of the information. In such a scenario, the knowledge of a genetic predisposition for autism may instead be a burden to the family and could also have negative implications for accessing other medical care through medical insurance-related policies and potentially lead to school and/or social discrimination. This conundrum is consistent with other potential biomarkers for autism including EEG, neuroimaging and behavioural observations—all of which are not as yet established for predicting autism likelihood in infancy.^36 37^

Second, the current landscape of autism treatment is fraught with limited access to intervention due to several factors including a lack of effective sustainable/low-intensity intervention programmes, trained therapists, financial limitations in accessing interventions and geographical factors precluding access.^38 39^ Effective interventions for autism^40^,^42^ do exist and lead to tangible gains in communication, cognitive and daily living skills in most children, but access to such interventions is a challenge in much of the world today. As such, providing timely, sustainable and effective interventions for children who are currently diagnosed with autism remains an immense challenge in most countries, both in the developed and developing world.^43^ Typical waitlists for autism treatments range from 1 to 2 years in most countries. Given this stark challenge, it is hard to posit that a healthcare system should and will be able to provide treatment for an infant or toddler who is at higher likelihood for autism based on a genetic test, in preference to older children who have been diagnosed and need treatment.

Third, current treatment options for autism entail behavioural intervention with positive but variable outcomes and do not cure or completely prevent the diagnosis.^41 42^ In other words, even with early and intensive behavioural intervention, it is not a given that the child will necessarily be completely well or not have any autism-related impairments in future. When coupled with the unpredictability of the exact phenotypic expression based on the genetic variant identified, this could result in an extremely stressful postnatal period for the family and caregivers of the infant in their attempts to process the information. In the scenario of an individual carrying a genetic risk variant but does not eventually manifest with the autism phenotype, knowledge of the genetic risk variant could do more harm than good.^33^ Identification of that specific genetic variant may violate the principle of non-maleficence by causing psychological harm to parents as explained above. Such information may also take resources and time away from the other siblings in the family and indirectly result in their detriment.

Fourth, the limitations of genetic testing to include autism-risk variants is important to highlight. This can lead to both false positive and false negative test results. False positives include the scenario where the presence of a genetic variant by itself may not necessarily result in the autistic phenotype. Given the current understanding of the aetiology of autism, and the proposed role of environmental factors, newborn genetic screens cannot take into account the environmental risk to the specific child. A result can thus be a false positive test, especially if the genetic variant by itself, without the environmental interaction, does not lead to the autism phenotype. This would mean that there is a much higher probability of developing autism based on the genetic result, but in reality, the likelihood is not elevated and is closer to the population baseline risk. On the other hand, a false negative test may arise where there is a missed detection of an at-risk genetic variant. This is because our current scientific knowledge related to autism is still limited and is rapidly evolving, hence there is the risk of tests not identifying (or reporting) certain genetic variants in a child because these may be currently classified as variants of uncertain significance (due to the knowledge base regarding their phenotypic association being limited). Similarly, genes which are not yet associated with autism in the scientific literature, at the point of testing would not be reported at all, although these may have an unidentified association. The proportion of genetic test results which are false negative or false positive as illustrated above, will likely change over time and likely reduce, in tandem with our growing understanding of the biological basis of autism. Hence, in future, with increased scientific literature, there may be a point in time where the ethical arguments for newborn genetic testing to include autism predisposition may change (especially if a specific panel of genetic variants are tested for) but for now, a normal newborn genetic screen in terms of autism predisposition would entail a risk reduction but not a risk elimination for autism and other related neurodevelopmental disorders. It is important for families to understand and contextualise this and this discussion will need to be done by a trained specialist.

Finally, the cost of genetic testing, especially at a population level and who should bear this is important to consider. When the likelihood for autism is not uniform across all individuals, with some groups being at higher likelihood compared with the general population (e.g., siblings of individuals on the autism spectrum) is it fair to subject all newborns to genetic testing that includes autism-risk variants? Or should this be something that is offered on an opt-in basis with the cost to be borne by the parents of at-risk newborns? In such a scenario, where financial subsidies are not provided, respecting the principle of parental autonomy will mean facilitating such testing; but with appropriate pretest and post-test counselling in place, especially to convey the nuances of phenotypic variance and limits of test result interpretation as discussed above. Conversely, it may be justifiable to offer such newborn genetic screening at a subsidised cost only to those families with newborns belonging to higher likelihood groups. Do the potential societal benefits from earlier detection of genetic risk for autism and subsequent pre-emptive treatment for the child justify the state or healthcare system bearing the costs—especially given the increasing prevalence of the condition? This argument related to cost of testing also needs to be considered in relation to the current situation whereby costs of genetic testing in general are reducing worldwide. The cost of genome sequencing has plummeted from the initial US$300 million in 1999–2000 to less than US$1000 today.^44^ Newborn genetic screening using genome sequencing is being evaluated in multiple sites.^45^,^47^ While the ethical arguments for such broad-based testing constitute another set of complex issues, in its implementation, there would be minimal added costs for inclusion of high penetrance autism genes and such a move could be justified. In such a scenario, it would also be important to then be prepared to disclose information regarding the presence of any high-penetrance genes associated with autism, with appropriate genetic counselling in place, so as to facilitate developmental surveillance and potentially timely intervention.

### Inclusiveness

The neurodiversity movement that recognises autism as a difference rather than a disability is also an important stakeholder in this debate. Increasingly, autistic individuals and parent advocates have questioned the overwhelmingly medical perspective of autism as a disorder, with historically accepted language at its core, when autism is an important identity for many individuals on the spectrum and often results in distinct strengths.^48^ Indeed, such advocates argue that using the neurotypical lens to view autism does gross injustice to autistic individuals and urge for greater participatory and inclusive research.^49^ Thus, the act of actively deciding against the birth of an individual with higher than population risk to be diagnosed on the autism spectrum may be perceived to go against the concepts of inclusiveness and embracing neurodiversity.

Related to the above, another important point to consider is the current dearth of studies examining the perspectives of autistic adults and parents of children who are on the spectrum, towards genetic testing for autism. It may well be that parents, based on their lived experiences, may choose against genetic testing for either their child on the spectrum or for future children. Alternatively, this decision-making may be based on specific factors or consequences of the additional knowledge gained by such testing. One such study was carried out by Johannessen *et al* in Norway examining caregiver attitudes in terms of opting for genetic testing.^50^ More than 75% of parents in that sample opined that they would opt for genetic testing if it could provide a genetic explanation for their child’s or their own autism. A similar proportion expressed that they would opt for such testing if the knowledge would make a difference to their child’s intervention timing and options. Interestingly, only 40% supported testing for the purpose of reproductive decision-making. In this sample, 66.8% were also concerned that increased genetic risk may lead to medical insurance-related discrimination and 56.1% felt that this may lead to unnecessary and increased concern over their child’s future. Other studies based on smaller samples have also suggested parents seeing benefits of genetic testing in terms of facilitating earlier access to intervention and explaining the aetiology of the condition.^51 52^ Varying perceptions of genetic testing based on the degree of impairments related to autism in their child has also been cited in another study, with caregivers of those with greater degree of features being more supportive.^53^ These studies in sum, illustrate how this is a complex topic and highlight the need for further qualitative exploration. Social factors such as culture, societal views and stigma and religious beliefs will also influence caregiver beliefs towards genetic testing. Hence targeted studies examining the views of the autistic community within each setting can reveal important preferences of critical stakeholders in this debate.

### An alternative proposition: diagnostic genetic testing for autism

Given the significant disadvantages and potential harm of newborn genetic screening, a middle ground may be instead to facilitate genetic testing for all children following a diagnosis of autism. This could be in the form of providing financially for families who are unable to afford such testing and especially where the results of such testing may influence parental reproductive decisions. This could also manifest in having the option available (in terms of service, logistics and personnel) within each healthcare setting for parents who may want to do such testing. This has the promise to be beneficial to the child, his/her parents as well as society in the following ways.

At the child level, identifying a specific genetic variant or cause for the child’s autism may help to refine treatment for related conditions. Some individuals have autism as a presentation of an inborn error of metabolism.^54^ Treatment of such conditions such as phenylketonuria, biotinidase deficiency, carnitine biosynthesis deficiency and urea cycle defects are readily available and can improve symptoms and prevent other clinical risks associated with the disorder. This applies especially to children with complex presentations and other medical symptoms which may not be readily explained. Identification of a specific genetic variant can thus directly impact clinical management of such children.

At the parental level, knowing a specific cause for their child’s autism can inform reproductive risk for parents and this information can be crucial in their decision for further children. Understanding specific risks, especially for high penetrance monogenic or chromosomal disorders, facilitates prenatal testing of an ongoing pregnancy or preimplantation genetic testing of embryos. This can allow the parents to exercise choice about future offspring. Being able to make an informed decision can be empowering to parents and respect their reproductive autonomy. This will be within the realm of procreative beneficence^55^ and important to consider as parents will bear the immediate and long-term burden of caring for a child on the autism spectrum. Some parents may opt to continue with a pregnancy where the fetus has been identified to carry an autism-risk variant. This still enables higher postnatal vigilance for the child’s developmental and behavioural needs to allow for intervention at the earliest possible juncture. For genetic variants with variable penetrance and phenotypic expression, it can be challenging for families to make reproductive decisions; support from an experienced genetic counsellor or geneticist will be invaluable.

Lastly, at the societal level, more widespread testing of children on the autism spectrum will enable more tailored treatment, better informed reproductive decision-making and earlier treatment, reducing costs to society. Further, it can potentially increase the scientific knowledge on the biological basis of autism and in turn, contribute to future advances in treatment in the field.

Indeed, from a distributive justice perspective, one can argue that every healthcare system should be able to provide genetic testing for autism. This includes being able to offer the tests, either locally or through international laboratories, having the necessary operational processes in place to run the tests and having trained healthcare professionals to counsel parents before and after genetic tests. Further, making genetic testing financially affordable or subsidised will be in the public interest in view of its impact not only on people on the autism spectrum, but also on their family, especially parents, and wider society.

## Back to the case

Let’s return to B’s case. The results of his genetic testing would have a significant chance of making a difference to J and P’s future reproductive decision-making. We believe that financial subsidies are justified. This would enable them to perform testing, thereby allowing their autonomy to be exercised. Their psychological well-being may potentially be improved by such information and this may have flow-on benefits for B. Such subsidies should be facilitated within the healthcare system, perhaps in conjunction with specific means of ascertaining financial capabilities so as to ensure that any couple who will benefit from such testing is able to access it.

## Data Availability

Data sharing is not applicable as no datasets were generated and/or analysed for this study. No data are available.
